# IgE, IgG4 and IgA specific to Bet v 1-related food allergens do not predict oral allergy syndrome

**DOI:** 10.1111/all.12534

**Published:** 2014-11-30

**Authors:** E E Guhsl, G Hofstetter, N Lengger, W Hemmer, C Ebner, R Fröschl, M Bublin, C Lupinek, H Breiteneder, C Radauer

**Affiliations:** 1 Department of Pathophysiology and Allergy Research, Medical University of ViennaVienna, Austria; 2 Floridsdorf Allergy CenterVienna, Austria; 3 Ambulatory for Allergy and Clinical ImmunologyVienna, Austria; 4 Department of Laboratory Medicine, Medical University of ViennaVienna, Austria

**Keywords:** Bet v 1, food allergy, IgA, IgE, IgG

## Abstract

**Background:**

Birch pollen-associated plant food allergy is caused by Bet v 1-specific IgE, but presence of cross-reactive IgE to related allergens does not predict food allergy. The role of other immunoglobulin isotypes in the birch pollen-plant food syndrome has not been investigated in detail.

**Methods:**

Bet v 1-sensitized birch pollen-allergic patients (*n* = 35) were diagnosed for food allergy by standardized interviews, skin prick tests, prick-to-prick tests and ImmunoCAP. Concentrations of allergen-specific IgE, IgG1, IgG4 and IgA to seven Bet v 1-related food allergens were determined by ELISA.

**Results:**

Bet v 1, Cor a 1, Mal d 1 and Pru p 1 bound IgE from all and IgG4 and IgA from the majority of sera. Immunoglobulins to Gly m 4, Vig r 1 and Api g 1.01 were detected in <65% of the sera. No significant correlation was observed between plant food allergy and increased or reduced levels of IgE, IgG1, IgG4 or IgA specific to most Bet v 1-related allergens. Api g 1-specific IgE was significantly (*P* = 0.01) elevated in celeriac-allergic compared with celeriac-tolerant patients. Likewise, frequencies of IgE (71% *vs* 15%; *P* = 0.01) and IgA (86% *vs* 38%; *P* = 0.04) binding to Api g 1.01 were increased.

**Conclusion:**

Measurements of allergen-specific immunoglobulins are not suitable for diagnosing Bet v 1-mediated plant food allergy to hazelnut and Rosaceae fruits. In contrast, IgE and IgA to the distantly related allergen Api g 1 correlate with allergy to celeriac.

About 70% of patients allergic to birch pollen (BP) show IgE-mediated reactions to plant foods such as hazelnut, apple, stone fruits, kiwi, carrot, celeriac and soya bean, typically mild reactions of the upper oral cavity, termed oral allergy syndrome (OAS) 1. These food allergies are a consequence of sensitization to Bet v 1 and subsequent IgE and T-cell cross-reactivity with homologous food allergens 2,3, such as Cor a 1.04 4, Mal d 1 5, Pru p 1 6, Api g 1 7,8 and Gly m 4 9. These allergens possess high sequence and structural identities with Bet v 1 10,11. Nearly all BP-allergic patients are sensitized to Bet v 1 12,13 and are at risk of developing plant food allergy 14. However, most patients show allergic reactions only to a limited number of potentially allergenic foods. Moreover, IgE specific to a certain Bet v 1-related allergen does not predict a clinically manifest allergy 15. The factors that determine the clinical spectrum of Bet v 1-associated plant food allergy are mostly unknown.

In contrast to IgE, Bet v 1-specific antibodies of other isotypes were less frequently investigated. Some patients undergoing allergen-specific immunotherapy (IT) develop blocking IgG which inhibits binding of allergens to IgE 16. Moreover, IgG4, IgG4/IgE ratios and IgG4 blocking activity were associated with tolerance to hazelnut and apple among Bet v 1-sensitized patients 13.

Few studies examining the role of allergen-specific IgA have been published. The levels of Bet v 1-specific IgA in nasal fluids of BP-allergic children increased during the pollen season, albeit with unclear clinical relevance 17. Examinations of the significance of IgA for the development of natural or induced tolerance in milk- or egg-allergic children showed no correlation 18,19.

Hence, we aimed to determine whether potentially tolerance-inducing allergen-specific IgG and IgA antibodies have an influence on the clinical activity of allergen-specific IgE. To this end, we used a panel of sera from well-characterized BP-allergic patients with individual patterns of plant food allergies to measure IgE, IgG1, IgG4 and IgA levels specific to Bet v 1-related allergens from hazelnut, peach, apple, soya, mung bean and celeriac. Moreover, we compared these concentrations with clinical symptoms to evaluate the significance of the different Ig classes in context with allergy or tolerance.

## Materials and methods

### Patients

Sera from 35 Bet v 1-sensitized BP-allergic patients were collected from two allergy outpatient clinics. BP allergy was diagnosed based on a convincing history of seasonal rhinoconjunctivitis or asthma and positive skin prick test (SPT) with BP extract (ALK-Abelló, Horsholm, Denmark). Bet v 1 sensitization was determined by ImmunoCAP (Thermo-Fisher, Uppsala, Sweden) with recombinant Bet v 1 (>0.35 kU_A_/l). Patients who had a positive SPT to mugwort pollen or were sensitized to profilin (determined by ImmunoCAP with recombinant Bet v 2 or SPT with natural Pho d 2) or Bet v 6 (determined by ImmunoCAP) were excluded.

Food allergies were diagnosed by standardized interviews performed by allergists using a questionnaire including 14 typical birch-associated foods (English translation in Supplementary Text S1). Patients were classified allergic to a specific food if they reported clear allergic symptoms (including OAS) after consumption of the food and tolerant if they had repeatedly consumed the raw food without experiencing adverse reactions. SPT and ImmunoCAP with commercial food extracts and prick-to-prick tests (PPT) with fresh foods or foods stored frozen until use were also conducted. Nine of the 35 patients had completed IT to BP or tree pollen mix.

Sera of 10 individuals without inhalative or food allergies were included as controls. The study was approved by the Ethics Committee of the Medical University of Vienna (approval number 718/2010). All patients provided written informed consent to their participation.

### Recombinant allergens

The cDNA of Pru p 1.0101 6 and codon-optimized synthetic genes (Eurofins MWG Operon, Ebersberg, Germany) of Bet v 1.0101, Cor a 1.0401, Mal d 1.0201, Gly m 4.0101, Vig r 1.0101, Api g 1.0101 and Api g 1.0201 (Supplementary Table S1) were ligated into the expression vector pET-28a(+) (Millipore, Darmstadt, Germany), expressed in *Escherichia coli* BL21[DE3] and purified as described previously 20. Identities, purities and native folds were confirmed by SDS-PAGE, matrix-assisted laser desorption ionization mass spectrometry and circular dichroism spectroscopy.

### Determination of allergen-specific Ig concentrations

Serum concentrations of allergen-specific IgE, IgG1, IgG4 and IgA were determined by a quantitative ELISA as described in Supplementary Text S2.

### Basophil activation assay

Basophil activation was measured using rat basophilic leukaemia cells that expressed the human FcεRI as described in Supplementary Text S2.

### Statistical evaluation

The Mann–Whitney *U*-test was used for comparing Bet v 1-specific Ig levels of patients with and without BP allergy, untreated and IT-treated patients and Ig levels of food-allergic and tolerant patients. Chi-squared tests were performed to compare frequencies of positive skin tests and allergen-specific Igs among food-allergic and tolerant patients. Potential diagnostic threshold values of food allergen-specific Igs were determined by receiver operator characteristic (ROC) curve analysis followed by chi-squared tests. *P*-values below 0.05 were considered significant. Analyses were performed with SPSS Statistics (IBM, Armonk, NY, USA) and GraphPad Prism (GraphPad Software, La Jolla, CA, USA).

## Results

### Food allergies

Nearly all patients (32/35; 91%) reported allergic reactions to plant foods, primarily to apple (30/35; 86%), stone fruits (26/35; 74%), hazelnut (25/35; 71%) and kiwifruit (16/35; 46%; Table1 and Supplementary Table S2). All 32 patients reported OAS, while 15 patients had experienced additional symptoms affecting the respiratory system in eight patients, the gastrointestinal tract in six, the eyes in four and the skin in four patients. Three patients reported anaphylaxis to walnut, soya milk and carrot, respectively. The nine patients who had undergone birch IT showed a similar spectrum of food allergies as the untreated patients (data not shown).

**Table 1:** Frequencies of plant food allergies (diagnosed by standardized interviews) and positive skin tests within the study population (*n* = 35)

	Questionnaire			SPT			PPT		
	Yes	No	n. c.	Pos.	Neg.	n. d.	Pos.	Neg.	n. d.
Apple	30	5	0	1	28	6	19	12	4
Stone fruits	26	9	0	1	33	1	27	7	1
Hazelnut	25	7	3	28	6	1	12	22	1
Celeriac	7	13	15	10	24	1	17	17	1
Soya bean/soya milk	6	14	15	3	30	2	17	16	2
Mung bean sprouts	1	27	7	0	0	35	15	18	2

n.c., not consumed; n.d., not done; PPT, prick-to-prick test; SPT, skin prick test.

### Allergen-specific Igs in BP-allergic and nonallergic patients

**Figure 1:**
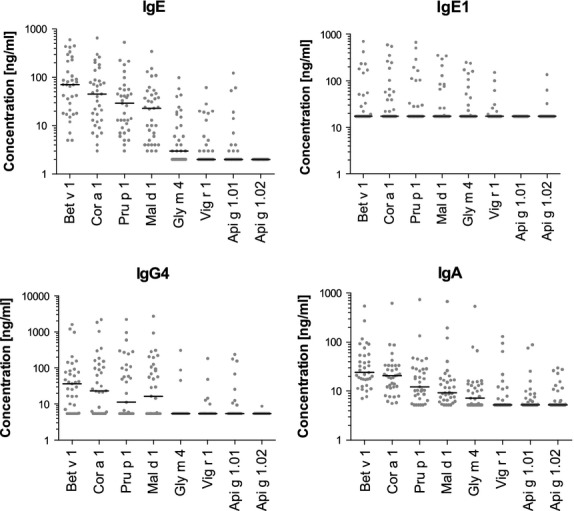
Individual levels of IgE, IgG1, IgG4 and IgA specific to Bet v 1-related allergens in sera from birch pollen-allergic patients (*n* = 35) measured by ELISA. Horizontal lines indicate medians calculated from all values. Negative values were set to the limits of detection (1.8 ng/ml for IgE, 17.2 ng/ml for IgG1, 5.4 ng/ml for IgG4 and 5.2 ng/ml for IgA).

Numbers of sera containing IgE, IgG1, IgG4 and IgA specific to the eight tested allergens and median antibody concentrations are shown in Table2. Distributions of allergen-specific Ig levels are displayed in Fig.1.

**Table 2:** Allergen-specific Ig recognition among birch pollen-allergic patients and nonallergic controls with median concentrations of allergen-specific Igs [ng/ml] calculated from positive values only

	IgE		IgG1		IgG4		IgA	
	Pos.	Median	Pos.	Median	Pos.	Median	Pos.	Median
**Birch pollen-allergic patients (*****n***** = 35)**								
Bet v 1	35	70	14	101	28	42	35	24
Cor a 1	35	45	16	62	24	81	35	21
Pru p 1	35	29	12	107	20	93	30	17
Mal d 1	35	23	12	85	20	89	31	12
Gly m 4	20	10	12	90	3	87	22	12
Vig r 1	15	6	7	31	5	14	12	12
Api g 1.01	11	7	0	–	8	47	16	8
Api g 1.02	0	–	3	63	1	9	15	11
**Nonallergic individuals (*****n***** = 10)**								
Bet v 1	0	–	0	–	0	–	5	8
Cor a 1	0	–	3	20	0	–	4	12
Pru p 1	1	2	1	60	0	–	4	19
Mal d 1	0	–	0	–	0	–	4	16
Gly m 4	0	–	2	144	0	–	4	13
Vig r 1	0	–	0	–	0	–	5	9
Api g 1.01	0	–	0	–	0	–	1	15
Api g 1.02	0	–	1	27	0	–	3	12

All sera from BP-allergic patients contained IgE specific to Bet v 1, Cor a 1, Mal d 1 and Pru p 1, whereas 31–57% showed IgE specific to Gly m 4, Vig r 1 and Api g 1.01. No serum contained Api g 1.02-specific IgE. Numbers of sera with IgE specific to Bet v 1-related food allergens and median IgE concentrations corresponded with per cent sequence identities to Bet v 1 (Supplementary Fig. S1).

Between 34% and 46% of the patients' sera contained IgG1 specific to Bet v 1, Cor a 1, Mal d 1, Pru p 1 and Gly m 4 with similar median concentrations. Contrary to the IgE binding pattern, no serum displayed IgG1 directed to Api g 1.01 while 9% bound to Api g 1.02. Nearly half of the sera (46%) lacked IgG1 against all tested allergens.

Numbers of sera containing allergen-specific IgG4 approached the numbers observed for IgE. When counting only positive results, median concentrations of IgG4 specific to Cor a 1, Pru p 1, Mal d 1 and Gly m 4 reached about twice the value of Bet v 1. Similarly to IgE, only a single serum showed IgG4 binding to Api g 1.02.

All sera contained IgA binding to Bet v 1 and Cor a 1. Percentages of positive values of the other tested allergens were similarly high as for IgE. Nearly half of the sera reacted with Api g 1.01 and Api g 1.02, respectively.

Patients who had undergone IT showed significantly elevated Bet v 1-specific IgG4 levels compared with untreated patients (median concentrations 83 and 27 ng/ml; *P* = 0.04; data not shown). No differences were observed for IgE, IgG1 and IgA.

**Figure 2:**
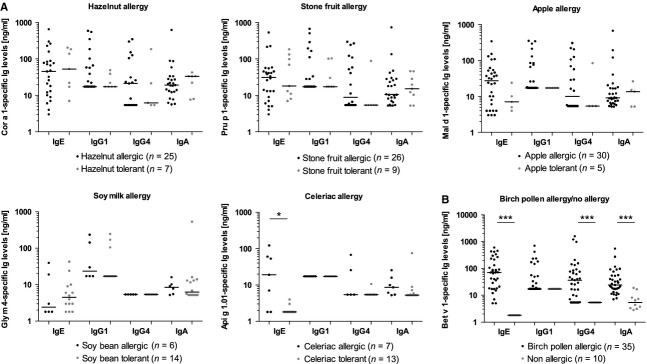
Distributions of food allergen-specific antibody levels [ng/ml] among birch pollen-allergic patients allergic or tolerant to the respective food (A) and Bet v 1-specific Ig levels of birch pollen-allergic *vs* nonallergic patients (B). Data of patients who had never consumed a specific food were excluded. Negative values were set to the limits of detection (1.8 ng/ml for IgE, 17.2 ng/ml for IgG1, 5.4 ng/ml for IgG4 and 5.2 ng/ml for IgA). *P < 0.05; ***P < 0.001.

None of the 10 nonallergic individuals' control sera contained IgE or IgG4 specific to any tested allergen with the exception of a single serum with marginally positive Pru p 1-specific IgE. Four sera had allergen-specific IgG1 to 1–3 food allergens. Eight sera contained IgA specific to 2–8 Bet v 1-related allergens. Nevertheless, Bet v 1-specific IgA levels were significantly higher in patients allergic to BP compared to nonallergic individuals (*P* < 0.001; Fig.2B).

### Comparing Ig levels with symptoms

A comparison of allergen-specific antibody levels of patients allergic or tolerant to hazelnut, apple, stone fruits, soya or celeriac is shown in Fig.2A. For IgG4, patients who had received BP IT were excluded to avoid biasing the analysis due to elevated IgG4 levels within that group.

No significant correlation between plant food allergy and increased or reduced levels of IgE, IgG1, IgG4 or IgA specific to the Bet v 1-related allergens was observed. In addition, IgG4/IgE and IgA/IgE ratios did not differ between food-allergic and tolerant patients. The only exception was Api g 1-specific IgE, which was significantly (*P* = 0.01) increased in celeriac-allergic patients (medians 19 ng/ml and <1.8 ng/ml).

### Diagnostic performance of skin tests, extract ImmunoCAP and Ig ELISA

Table3 summarizes the diagnostic sensitivities and specificities of skin tests and allergen-specific Ig measurements when comparing food-allergic and food-tolerant patients. Sensitivities of SPT were low (4–57%) with the exception of hazelnut (83%). Consequently, specificities were only 14% for hazelnut, but 100% for the other tested extracts. In contrast, sensitivities of PPT were considerably higher (63–100%) with the exception of hazelnut with only 33%. We observed significantly increased frequencies of positive celeriac SPT (57% *vs* 0%; *P* = 0.002) and PPT (100% *vs* 15%; *P* < 0.001) among celeriac-allergic patients as well as positive soya milk PPT (100% *vs* 29%; *P* = 0.01) among soya allergic patients.

**Table 3:** Diagnostic performance of skin prick test (SPT), prick-to-prick test (PPT), ImmunoCAP and allergen-specific Ig (positive/total)

Allergy	SPT	PPT	CAP	IgE	IgG1	IgG4	IgA
**Hazelnut allergy**	Hazelnut			Cor a 1			
	*n* = 31	*n* = 31	*n* = 32	*n* = 32			
Allergic	20/24	8/24	24/25	25/25	11/25	17/25	25/25
Tolerant	6/7	3/7	7/7	7/7	3/7	5/7	7/7
Sensitivity [%]	83	33	96	100	44	68	100
Specificity [%]	14	57	0	0	57	29	0
**Stone fruit allergy**	Peach			Pru p 1			
	*n* = 34	*n* = 34	*n* = 35	*n* = 35			
Allergic	1/25	19/25	21/26	26/26	9/26	17/26	23/26
Tolerant	0/9	8/9	8/9	9/9	3/9	3/9	7/9
Sensitivity [%]	4	76	81	100	35	65	88
Specificity [%]	100	11	11	0	67	67	22
**Apple allergy**	Apple			Mal d 1			
	*n* = 29	*n* = 31	*n* = 35	*n* = 35			
Allergic	1/25	17/27	20/30	30/30	12/30	18/30	28/30^*^
Tolerant	0/4	2/4	3/5	5/5	0/5	2/5	3/5^*^
Sensitivity [%]	4	63	67	100	40	60	93
Specificity [%]	100	50	40	0	100	60	40
**Soya milk allergy**	Soya bean			Gly m 4			
	*n* = 18	*n* = 18	*n* = 20	*n* = 20			
Allergic	1/4	4/4^*^	0/6	3/6	3/6	0/6	5/6
Tolerant	0/14	4/14^*^	0/14	11/14	3/14	0/14	8/14
Sensitivity [%]	25	100	0	50	50	0	83
Specificity [%]	100	71	100	21	79	100	43
**Celeriac allergy**	Celeriac			Api g 1.01			
	*n* = 20	*n* = 20	*n* = 20	*n* = 20			
Allergic	4/7^*^^*^	7/7^*^^*^^*^	6/7	5/7^*^	0/7	3/7	6/7^*^
Tolerant	0/13^*^^*^	2/13^*^^*^^*^	8/13	2/13^*^	0/13	2/13	5/13^*^
Sensitivity [%]	57	100	86	71	0	43	86
Specificity [%]	100	85	38	85	100	85	62
				Api g 1.02			
Allergic				0/7	1/7	0/7	3/7
Tolerant				0/13	1/13	1/13	7/13
Sensitivity [%]				0	14	0	43
Specificity [%]				100	92	92	46

Significant differences between allergic and tolerant patients are marked by asterisks (^*^P < 0.05; ^*^^*^P < 0.01; ^*^^*^^*^P < 0.001).

No connections between extract ImmunoCAP results and food allergy were detected. Hazelnut and peach ImmunoCAP showed high sensitivities but low specificities with positive results with nearly all sera of food-allergic and tolerant patients. In contrast, the soya bean ImmunoCAP was negative with all sera.

Measurements of allergen-specific Ig yielded similar recognition frequencies in allergic and tolerant patients with high sensitivities but low specificities for IgE and IgA. Mal d 1-specific IgA was found with significantly higher frequency among apple-allergic than apple-tolerant patients (93% *vs* 60%, *P* = 0.03). Celeriac-allergic patients showed increased frequencies of IgE (71% *vs* 15%; *P* = 0.01) and IgA (86% *vs* 38%; *P* = 0.04) to Api g 1.01 (Table3). When applying threshold levels obtained by ROC analyses, significantly different frequencies were observed for IgE, IgG4 and IgA to Api g 1.01 (Supplementary Table S3). No Ig thresholds for the other allergens could be established.

### Basophil degranulation assay

**Figure 3:**
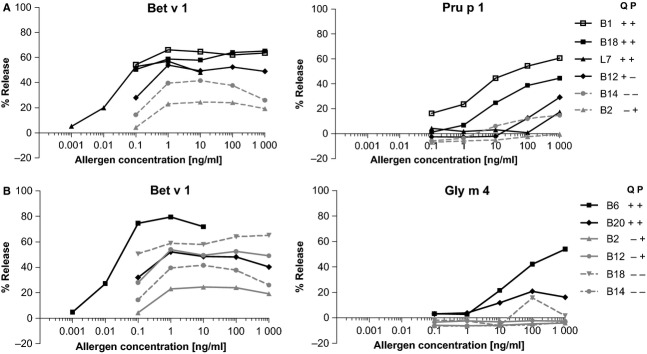
Basophil degranulation assay. Rat basophilic leukemia cells were sensitized with birch pollen-allergic patients' sera allergic or tolerant to peach (A) or soya milk (B) and activated by Bet v 1, Pru p 1 and Gly m 4. Q: allergy (+) or tolerance (−) according to the questionnaire; P: positive (+) or negative (−) prick-to-prick test to peach (A) or soya milk (B).

A comparison of the occurrence of peach and soya allergy with the results of a basophil activation assay is shown in Fig.3 for six patients each. All tested sera elicited degranulation by Bet v 1 with maximum hexosaminidase releases at 1 ng/ml Bet v 1 (Fig.3). Sensitization of the cells by all four tested sera of peach-allergic patients and one of the two sera of peach-tolerant patients induced Pru p 1-mediated degranulation, albeit to a slightly lower extent and at considerably higher allergen concentrations (at least 1000-fold) compared with Bet v 1 (Fig.3A). Both sera of soya-allergic patients activated the basophils upon incubation with Gly m 4, while cells sensitized with three of the four soya-tolerant patients' sera did not react with Gly m 4 (Fig.3B).

## Discussion

More than 90% of BP-allergic patients are sensitized to Bet v 1, the causative agent of most cases of BP-associated plant food allergy 13. However, sensitization to Bet v 1 usually does not trigger allergic reactions to all foods that contain Bet v 1-related allergens. Currently, no diagnostic tests other than double-blind placebo-controlled food challenge (DBPCFC) are available to reliably predict allergic reactions to specific plant foods. However, DBPCFC is time-consuming and has its own problems such as lack of standardization, lability of allergens, difficulties in blinding and variability of the patients' susceptibility and is not suitable for routine use 21. Hence, in our study, we used questionnaire-based interviews performed by allergists in specialized outpatient clinics to diagnose BP-associated food allergy. Skypala et al. 22 showed that a simple questionnaire-based diagnosis of pollen-related food allergy yielded results highly similar to those of a protocol combining clinical history, PPT and DBPCFC.

We compared IgE levels to Bet v 1 and Bet v 1-related food allergens in a group of BP-allergic patients with varying profiles of food allergies. We detected a correlation between IgE to a Bet v 1-related allergen and allergic symptoms to the respective source only for celeriac allergy and Api g 1.01, both for IgE levels and frequencies (Fig.2A, Table3). These results are in line with previous data from studies of patients allergic to hazelnut 23, apple 15 and kiwifruit 24 showing similar frequencies of sensitization to Cor a 1, Mal d 1 and Act d 8 in food-allergic and food-tolerant BP-allergic patients. In contrast, BP-allergic patients allergic to celeriac 25 and carrot 26 showed considerably higher frequencies of IgE binding to Api g 1 and Dau c 1 than tolerant patients. Likewise, ImmunoCAP with food extracts (including celeriac) did not discriminate between food-allergic and tolerant patients (Table3).

The frequency of IgG4 binding to Bet v 1-related allergens, allergen-specific IgG4 levels and IgG4/IgE ratios were similar for food-allergic and tolerant patients (Fig.2A, Table3), corroborating the lacking correlation between food allergy or tolerance and allergen-specific IgG4 27. In contrast, Geroldinger-Simic et al. 13 found elevated food allergen-specific IgG4/IgE ratios in hazelnut- or apple-tolerant BP-allergic patients, while tolerant patients from our study population did not display significantly increased levels of Cor a 1 or Mal d 1-specific IgG4 (Fig.2A).

A high percentage of both BP-allergic and nonallergic individuals had low levels of IgA specific to Bet v 1-related allergens (Table2). The role of IgA in allergy and its possible protective effect are controversial. Children allergic to milk or egg did not show decreased allergen-specific IgA levels compared with tolerant children 18,19. However, low milk-specific and mite-specific IgA was associated with persistence of milk allergy 28 and occurrence of house dust mite allergy in children 29. In our study, however, patients allergic to apple, soya and celeriac showed higher frequencies of IgA specific to Mal d 1, Gly m 4 and Api g 1.01, respectively, than tolerant patients (Table3).

Frequencies of IgE, IgG4 and IgA binding to Cor a 1, Mal d 1 and Pru p 1 were similar with nearly all patients' sera displaying Ig binding. In contrast, Gly m 4, Vig r 1 and Api g 1.01, possessing <50% sequence identity to Bet v 1 and hence being less cross-reactive, were recognized by a minority of the sera with lower amounts of bound Ig (Table2). Patient-specific patterns of epitope recognition determine Ig binding to these distantly related allergens and may be clinically relevant as indicated by significantly increased frequencies of Api g 1.01-specific IgE, IgG4 and IgA among celeriac-allergic patients (Table3, Table S3).

Responses to Bet v 1-related food allergens in the basophil degranulation assay showed a high extent of agreement with reported food allergy (Fig.3). All peach- and soya-allergic patients' sera induced degranulation after activation by Pru p 1 or Gly m 4, respectively, while only two of six food-tolerant patients reacted to the respective allergen. Basophil activation tests with extracts from hazelnut, apple, celeriac and kiwifruit were previously shown to possess high sensitivities and specificities in discriminating food-allergic from tolerant BP-allergic patients 15,30. Comparing skin tests with symptoms, we observed high sensitivities but low specificities for PPT with fresh fruits and low sensitivities for SPT with commercial extracts with the exception of hazelnut, corroborating previous results 24,30. This is explained by the instability of Bet v 1-related allergens, which are destroyed during extract preparation unless using specialized procedures 31. Lower sensitivities were also observed for ImmunoCAPs with extracts compared with IgE-ELISAs with the corresponding recombinant allergens (Table3). The only exception was the hazelnut ImmunoCAP, which is spiked with recombinant Cor a 1 to enhance its sensitivity for detecting BP-associated hazelnut allergy 32.

In summary, we conclude that levels of allergen-specific Igs show no connection with Bet v 1-associated plant food allergy to hazelnut and Rosaceae fruits. In contrast, IgE and possibly IgA to more distantly related allergens may to a certain extent predict allergy to foods such as soya milk and celeriac. Furthermore, our data do not support a protective role of IgA and IgG4.

## Abbreviations

BP: birch pollen
DBPCFC: double-blind placebo-controlled food challenge
Ig: immunoglobulin
IT: immunotherapy
PPT: prick-to-prick test
SPT: skin prick test

## Supporting Information

**Figure S1.** Comparison of Ig binding of Bet v 1-related food allergens with their sequence identities to Bet v 1.

**Table S1.** Recombinant allergens used in this study.

**Table S2.** Additional allergic reactions to raw foods included in the questionnaire, reported by birch pollen allergic patients (*n* = 35).

**Table S3.** Diagnostic performance of thresholds in allergen-specific Ig determination.

**Text S1.** English translation of the questionnaire used in interviews of the study patients.
