# Supplementary material for: IgE, IgG4 and IgA specific to Bet v 1-related food allergens do not predict oral allergy syndrome
Source: Allergy. 2014 Nov 30;70(1):59–66. doi: 10.1111/all.12534 (PMC4283702; doi:10.1111/all.12534)
Supplement: Supplementary file 1 — Figure S1. Comparison of Ig binding of Bet v 1-related food allergens with their sequence identities to Bet v 1. [file all0070-0059-sd1.doc]

**Supplementary figure S1**

**Fig. S1. Comparison of Ig binding of Bet v 1-related food allergens with their sequence identities to Bet v 1.** A. Correlation of Ig binding to Bet v 1 with Ig binding to Bet v 1-related food allergens; B. Frequencies of Ig binding to Bet v 1-related food allergens among birch pollen-allergic patients (n = 35).
