# Supplementary material for: IgE, IgG4 and IgA specific to Bet v 1-related food allergens do not predict oral allergy syndrome
Source: Allergy. 2014 Nov 30;70(1):59–66. doi: 10.1111/all.12534 (PMC4283702; doi:10.1111/all.12534)
Supplement: Supplementary file 2 — Table S1. Recombinant allergens used in this study. [file all0070-0059-sd2.doc]

**Supplementary table S1.**

**Table S1.** Recombinant allergens used in this study

| **Allergen** | **Source** | **Uniprot accession number** | **% Sequence identity to Bet v 1** |
| --- | --- | --- | --- |
| Bet v 1.0101 | birch pollen | P15494 | 100 |
| Cor a 1.0401 | hazelnut | Q9SWR4 | 68 |
| Pru p 1.0101 | peach | Q2I6V8 | 59 |
| Mal d 1.0201 | apple | Q9S7M5 | 57 |
| Gly m 4.0101 | soy bean | P26987 | 47 |
| Vig r 1.0101 | mung bean | Q2VU97 | 45 |
| Api g 1.0101 | celeriac | P49372 | 42 |
| Api g 1.0201 | celeriac | P92918 | 40 |
