# Supplementary material for: IgE, IgG4 and IgA specific to Bet v 1-related food allergens do not predict oral allergy syndrome
Source: Allergy. 2014 Nov 30;70(1):59–66. doi: 10.1111/all.12534 (PMC4283702; doi:10.1111/all.12534)
Supplement: Supplementary file 3 — Table S2. Additional allergic reactions to raw foods included in the questionnaire, reported by birch pollen allergic patients (n = 35). [file all0070-0059-sd3.doc]

**Supplementary table S2.**

**Table S2.** Additional allergic reactions to raw foods included in the questionnaire, reported by birch pollen allergic patients (n=35).

|  | **Questionnaire** | | |
| --- | --- | --- | --- |
|  | **yes** | **no** | **n. c.** |
| Kiwi fruit | 16 | 16 | 3 |
| Walnut | 15 | 14 | 6 |
| Pear | 13 | 18 | 4 |
| Fig | 12 | 12 | 11 |
| Almond | 11 | 19 | 5 |
| Peanut | 10 | 22 | 3 |
| Persimmon | 2 | 18 | 15 |
| Tofu | 1 | 19 | 15 |

n.c.: not consumed
