# Supplementary material for: IgE, IgG4 and IgA specific to Bet v 1-related food allergens do not predict oral allergy syndrome
Source: Allergy. 2014 Nov 30;70(1):59–66. doi: 10.1111/all.12534 (PMC4283702; doi:10.1111/all.12534)
Supplement: Supplementary file 4 — Table S3. Diagnostic performance of thresholds in allergen-specific Ig determination. [file all0070-0059-sd4.doc]

**Supplementary table S3**

**Table S3.** Diagnostic performance of thresholds in allergen-specific Ig determination. Threshold concentrations were determined by ROC analysis. P-values refer to chi-squared tests.

| **Allergen** | **Isotype** | **Threshold [ng/ml]** | **Sensitivity [%]** | **Specificity [%]** | **p** |
| --- | --- | --- | --- | --- | --- |
| Api g 1.01 | IgE | 5.5 | 71 | 100 | 0.0004 |
| Api g 1.01 | IgG4 | 19.0 | 43 | 100 | 0.01 |
| Api g 1.01 | IgA | 6.0 | 71 | 77 | 0.04 |
