# Supplementary material for: IgE, IgG4 and IgA specific to Bet v 1-related food allergens do not predict oral allergy syndrome
Source: Allergy. 2014 Nov 30;70(1):59–66. doi: 10.1111/all.12534 (PMC4283702; doi:10.1111/all.12534)
Supplement: Supplementary file 5 — Text S1. English translation of the questionnaire used in interviews of the study patients. [file all0070-0059-sd5.doc]

**Supplementary text S1.** English translation of the questionnaire used in interviews of the study patients.

**1. Patient’s data**

Code: ___________

Gender: male ⬜ female ⬜

Year of birth: ____________

**2. Inhalative allergy**

Symptoms:

⬜ Rhinoconjunctivitis

Duration of symptoms:

⬜ perennial

⬜ January ⬜ February ⬜ March ⬜ April

⬜ May ⬜ June ⬜ July ⬜ August

⬜ Sept. ⬜ Oct. ⬜ Nov. ⬜ Dec.

⬜ Asthma

Duration of symptoms:

⬜ perennial

⬜ January ⬜ February ⬜ March ⬜ April

⬜ May ⬜ June ⬜ July ⬜ August

⬜ Sept. ⬜ Oct. ⬜ Nov. ⬜ Dec.

In which year have allergic symptoms appeared first? _____________

Did you undergo allergen-specific immunotherapy? ⬜ yes ⬜ no

Against which allergens? ______________________________________________

Duration from ____________ to ____________________

Do you take anti-allergic medication?

⬜ yes; which one? ______________________________________________________

⬜ no

3**. Food allergy**

⬜ yes ⬜ no

Foods:

| **Food** | **Allergic reactions** | **Symptoms** | **Year of first occurrence** |
| --- | --- | --- | --- |
| Apple | ⬜ yes ⬜ no ⬜ not consumed  ⬜ raw ⬜ processed |  |  |
| Pear | ⬜ yes ⬜ no ⬜ not consumed  ⬜ raw ⬜ processed |  |  |
| Peach/Nectarine | ⬜ yes ⬜ no ⬜ not consumed  ⬜ raw ⬜ processed |  |  |
| Cherry | ⬜ yes ⬜ no ⬜ not consumed  ⬜ raw ⬜ processed |  |  |
| Apricot | ⬜ yes ⬜ no ⬜ not consumed  ⬜ raw ⬜ processed |  |  |
| Plum | ⬜ yes ⬜ no ⬜ not consumed  ⬜ raw ⬜ processed |  |  |
| Kiwifruit | ⬜ yes ⬜ no ⬜ not consumed  ⬜ raw ⬜ processed |  |  |
| Almond | ⬜ yes ⬜ no ⬜ not consumed  ⬜ raw ⬜ processed |  |  |
| Hazelnut | ⬜ yes ⬜ no ⬜ not consumed  ⬜ raw ⬜ processed |  |  |
| Walnut | ⬜ yes ⬜ no ⬜ not consumed  ⬜ raw ⬜ processed |  |  |
| Celery/celeriac | ⬜ yes ⬜ no ⬜ not consumed  ⬜ raw ⬜ processed |  |  |
| Carrot | ⬜ yes ⬜ no ⬜ not consumed  ⬜ raw ⬜ processed |  |  |
| Soy milk | ⬜ yes ⬜ no ⬜ not consumed  ⬜ raw ⬜ processed |  |  |
| Tofu | ⬜ yes ⬜ no ⬜ not consumed  ⬜ raw ⬜ processed |  |  |
| Mung bean sprouts | ⬜ yes ⬜ no ⬜ not consumed  ⬜ raw ⬜ processed |  |  |
| Peanut | ⬜ yes ⬜ no ⬜ not consumed  ⬜ raw ⬜ processed |  |  |
| Fig | ⬜ yes ⬜ no ⬜ not consumed  ⬜ raw ⬜ processed |  |  |
| Persimmon | ⬜ yes ⬜ no ⬜ not consumed  ⬜ raw ⬜ processed |  |  |
| Other: |  |  |  |
|  | ⬜ yes ⬜ no ⬜ not consumed  ⬜ raw ⬜ processed |  |  |
|  | ⬜ yes ⬜ no ⬜ not consumed  ⬜ raw ⬜ processed |  |  |
|  | ⬜ yes ⬜ no ⬜ not consumed  ⬜ raw ⬜ processed |  |  |
