# Supplementary material for: IgE, IgG4 and IgA specific to Bet v 1-related food allergens do not predict oral allergy syndrome
Source: Allergy. 2014 Nov 30;70(1):59–66. doi: 10.1111/all.12534 (PMC4283702; doi:10.1111/all.12534)
Supplement: Supplementary file 6 — Text S2. Supplementary materials and methods. [file all0070-0059-sd6.doc]

**Supplementary text S2**

**Materials and methods**

**Determination of allergen-specific immunoglobulins in patients’ sera**

MaxiSorp 96-well plates (Nunc, Roskilde, Denmark) were coated with 1 µg/ml allergen in 50 mM NaHCO_3_ (pH 9.5) at 4°C over night. Blocking was performed with Superblock Blocking Buffer (Pierce, Thermo Scientific, Waltham, MA, USA) for measuring IgE and IgG4 or with Tris-buffered saline containing 0.5% Tween-20 (TBST) with 3% bovine serum albumin (BSA) for IgG1 and IgA for 2 h at room temperature (RT). Sera from allergic patients or non-allergic individuals were diluted 1:10-1:120 (IgE), 1:100 (IgG1, IgA) or 1:200 (IgG4) in TBST with 0.5% BSA and applied in duplicates over night. Bound Igs were detected with alkaline phosphatase conjugated anti-human IgE, IgG1, IgG4 (BD Pharmingen, Heidelberg, Germany), or IgA (Sigma-Aldrich, St. Louis, MO, USA) and p-nitrophenyl phosphate (Sigma-Aldrich). Colour development was measured at 405 nm with 550 nm as reference wavelength.

Quantitation was performed by using standard curves of human IgA, IgG1 and IgG4 (Calbiochem, Merck Millipore, Billerica, MA, USA). As catching antibodies for the standards, mouse anti-human IgA1/IgA2 and anti-human IgG (BD Pharmingen) were applied. Quantitation of IgE was performed using a recombinant chimeric Bet v 1-specific IgE derived from the mouse anti-Bet v 1 monoclonal antibody BIP1 and Bet v 1 as catching protein. For Bet v 1-specific IgE, concentrations determined by this ELISA matched those from the ImmunoCAP (linear correlation coefficient 0.94; data not shown). The limits of detection, defined as the standard concentrations yielding OD-values exceeding the buffer controls by more than three standard deviations, were 1.8 ng/ml for IgE, 17.2 ng/ml for IgG1, 5.4 ng/ml for IgG4 and 5.2 ng/ml for IgA considering serum dilutions. Only serum Ig-concentrations exceeding these values were considered positive.

## Basophil activation assay

Rat basophilic leukaemia cells RS-ATL8 expressing the human FcεRI (kindly provided by Ryosuke Nakamura, National Institute of Health Sciences, Tokyo, Japan) were plated into 96 well polystyrene cell culture plates (Corning Inc., Corning, NY, USA) and incubated with sera diluted 1:10-1:40 in minimum essential medium containing 10% foetal calf serum (both from Gibco, Life Technologies, Carlsbad, CA, USA) at 37°C over night. Tenfold serial dilutions of allergens (0.001 ng/ml to 1 µg/ml) were added for 1 h. Degranulation was measured in the supernatants as β-hexosaminidase release using the fluorescent substrate 4-methylumbelliferyl-N-acetyl-β-D-galactosaminide (Sigma-Aldrich, St. Louis, MO, USA) at 355 nm excitation and 460 nm detection wavelengths. Percent activation was calculated relative to hexosaminidase content of cells lysed with triton X-100 (100% release) and the supernatant of untreated cells (0% release).

**Reference**

1. Laffer S, Vangelista L, Steinberger P, Kraft D, Pastore A, Valenta R. Molecular characterization of Bip 1, a monoclonal antibody that modulates IgE binding to birch pollen allergen, Bet v 1. *J Immunol* 1996;**157**(11):4953-4962.

2. Reginald K, Eckl-Dorna J, Zafred D, Focke-Tejkl M, Lupinek C, Niederberger V, et al. Different modes of IgE binding to CD23 revealed with major birch allergen, Bet v 1-specific monoclonal IgE. *Immunol Cell Biol* 2013;**91**:167-172.

3. Nakamura R, Uchida Y, Higuchi M, Tsuge I, Urisu A, Teshima R. A convenient and sensitive allergy test: IgE crosslinking-induced luciferase expression in cultured mast cells. *Allergy* 2010;**65**:1266-1273.

4. Nakamura R, Ishiwatari A, Higuchi M, Uchida Y, Kawakami H, Urisu A, et al. Evaluation of the luciferase assay-based in vitro elicitation test for serum IgE. *Allergol Int* 2012;**61**:431-437.
